# OFF-State Leakage Suppression in Vertical Electron–Hole Bilayer TFET Using Dual-Metal Left-Gate and N^+^-Pocket

**DOI:** 10.3390/ma15196924

**Published:** 2022-10-06

**Authors:** Hu Liu, Wenting Zhang, Zaixing Wang, Yao Li, Huawei Zhang

**Affiliations:** 1 School of Electronic and Information Engineering, Lanzhou Jiaotong University, Lanzhou 730070, China; 2 Key Laboratory of Opto-Technology and Intelligent Control, Ministry of Education, Lanzhou Jiaotong University, Lanzhou 730070, China

**Keywords:** tunnel field-effect transistor, OFF-state leakage suppression, electron–hole bilayer, line tunneling

## Abstract

In this paper, an In_0.53_Ga_0.47_As electron–hole bilayer tunnel field-effect transistor (EHBTFET) with a dual-metal left-gate and an N^+^-pocket (DGNP-EHBTFET) is proposed and systematically studied by means of numerical simulation. Unlike traditional transverse EHBTFETs, the proposed DGNP-EHBTFET can improve device performance without sacrificing the chip density, and can simplify the manufacturing process. The introduction of the dual-metal left-gate and the N^+^-pocket can shift the point-tunneling junction and adjust the energy band and the electric field in it, aiming to substantially degrade the OFF-state current (*I*_OFF_) and maintain good ON-state performance. Moreover, the line tunneling governed by the tunneling-gate and the right-gate can further regulate and control *I*_OFF_. By optimizing various parameters related to the N^+^-pocket and the gate electrodes, DGNP-EHBTFET’s *I*_OFF_ is reduced by at least four orders of magnitude, it has a 75.1% decreased average subthreshold swing compared with other EHBTFETs, and it can maintain a high ON-state current. This design greatly promotes the application potential of EHBTFETs.

## 1. Introduction

As the feature size enters the nanoscale and continues to shrink, the increasing static power consumption in metal-oxide-semiconductor field-effect transistors (MOSFETs) places restrictions on the development of integrated circuits. To continue Moore’s law, the static power consumption of devices must be further reduced. Limited by the injection mechanism of thermal emission for MOSFETs, an effective method to solve this problem is to develop steep subthreshold swing (*SS*) devices. One of the mainstream switching devices with steep *SS*, the tunneling field-effect transistor (TFET) [1,2,3], has the potential to achieve a low operating voltage by overcoming the thermally limited *SS* of 60 mV/decade by utilizing tunneling as a switching mechanism, and has attracted extensive attention due to its low-power-consumption feature. Si-based TFETs can take advantage of the existing semiconductor design and manufacturing infrastructure, and has thus become the research mainstream of TFETs. However, a low ON-state current (*I*_ON_) caused by the inherent material properties of silicon restricts the practical commercial use of TFETs. Therefore, various TFETs with novel materials or structures have emerged to improve *I*_ON_, such as nanowire TFETs [4,5,6], heterojunction TFETs [6,7,8,9,10], dopingless and junctionless TFETs [4,9,11,12,13,14,15], line-tunneling (LT) TFETs [15,16,17,18,19,20,21,22,23], two-dimensional material TFETs [24,25,26], etc. Among them, an electron–hole bilayer TFET (EHBTFET) [15,18,19,20,21,22,23] based on LT can boost *I*_ON_ due to having a larger tunneling region, and has been developed in recent years.

To conserve the total momentum in tunneling, III-V direct band gap materials do not have to involve phonons, which is more beneficial to the improvement of TFET performance than an indirect band gap semiconductor material such as silicon. By analyzing TFETs related to III-V materials in previous publications [9,11,14], it is confirmed that InGaAs is one of the candidate materials suitable for the design of N-type TFETs. In particular, In_0.53_Ga_0.47_As has a low effective electron mass, a narrow band gap, ultra-high electron mobility, and lattice matching with InP substrate, which has been widely used in the low-power-consumption applications [27,28]. Similarly, it can be employed as the bulk material of EHBTFETs to further improve *I*_ON_ [29,30]. Nevertheless, the band-to-band tunneling leakage in the OFF-state caused by point tunneling (PT) in In_0.53_Ga_0.47_As-based EHBTFETs can result in a higher OFF-state current (*I*_OFF_) compared with Si-based ones, which will increase *SS* so as to increase the static power consumption of devices. To circumvent this issue, Alper et al. [31] have proposed a novel EHBTFET where counterdoping is carried out in the underlap regions. However, to maintain the same *I*_ON_, the gate voltage (*V*_gs_) needs to be increased to 2.6 V, which is unfavorable for low-power-consumption applications. To obtain low *I*_OFF_, another technique [32] can be employed, that is, the high-K dielectric pocket is inserted into the underlap regions near the source and the drain regions, but it is only applicable to EHBTFETs with a transverse structure. The *I*_ON_ of transverse EHBTFETs directly depends on the gate overlap area, which will restrict the reduction in the lateral size of the device. In addition, the manufacturing process of transverse EHBTFETs is complicated, which will cause a reduction in the product yield rate in fabrication.

In this paper, an In_0.53_Ga_0.47_As EHBTFET with a dual-metal left-gate and an N^+^-pocket (DGNP-EHBTFET) is proposed to effectively solve the aforementioned problem. The proposed DGNP-EHBTFET has a vertical structure, which can improve *I*_ON_ by enlarging the gate overlap region in the vertical direction without sacrificing the chip density. Meanwhile, the realization of the asymmetric double-gate structure in DGNP-EHBTFET does not need the transfer substrate technique used in transverse EHBTFETs, thereby simplifying the manufacturing process. The introduction of the dual-metal left-gate can not only make the PT junction deviate from the gate overlap region, but also adjust the electric field profiles at the tunneling junction, both of which are conducive to reducing *I*_OFF_. The N^+^-pocket inserted into the gate underlap region near the drain can regulate and control the tunneling energy range and the tunneling distance of PT, which is beneficial to further decreasing *I*_OFF_ but can maintain a good *I*_ON_. Moreover, *I*_OFF_ is also affected by LT that is governed by the work-function of the tunneling-gate and the right-gate. Until now, since there are relatively few studies on the OFF-state leakage suppression of vertical EHBTFETs, it is necessary for the device mechanism of the proposed novel structure to be revealed.

This paper is organized as follows. Section 2 introduces the device structures, the related physical parameters and models, and the manufacturing process. The operating mechanism, the direct current (DC) characteristics for the three EHBTFETs, as well as the effects of the N^+^-pocket and the work-function of the gate electrodes on DGNP-EHBTFET are thoroughly investigated in Section 3. Finally, Section 4 briefly summarizes the present studies.

## 2. Devices Structure and Simulation Methods

**Figure 1:**
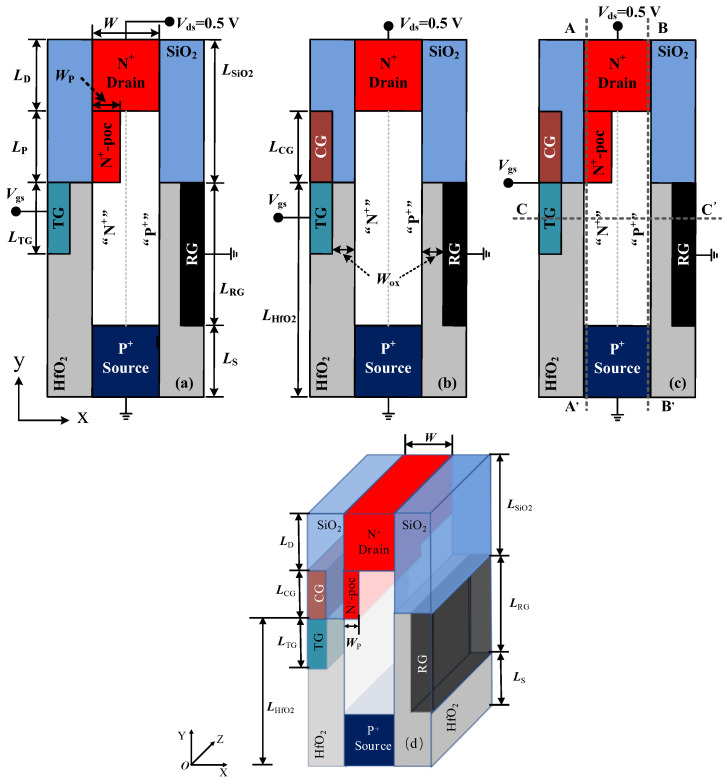
Cross sectional views of (**a**) NP-EHBTFET; (**b**) DG-EHBTFET; and (**c**) DGNP-EHBTFET. (**d**) A 3D schematic of DGNP-EHBTFET. Cutlines A–A’ and B–B’ are located at 0.5 nm on the right side of the left-gate dielectric and the left side of the right-gate dielectric, respectively, and cutline C–C’ is located in the middle of the tunneling-gate.

To better reveal the device mechanism of the proposed DGNP-EHBTFET, two other EHBTFETs are introduced in this paper: (1) an In_0.53_Ga_0.47_As EHBTFET with a dual-metal left-gate (DG-EHBTFET) and (2) an In_0.53_Ga_0.47_As EHBTFET with an N^+^-pocket (NP-EHBTFET). Cross-sectional views of these three vertical-structure EHBTFETs and a 3D schematic of DGNP-EHBTFET are shown in Figure 1, and the corresponding structural parameters are listed in Table 1.

For these three EHBTFETs, the structural parts are the same except for the left-gate and the channel region connected to the drain region. The left-gates of DG-EHBTFET and DGNP-EHBTFET are composed of a tunneling-gate (TG) and a control-gate (CG) tied directly, while that of NP-EHBTFET is only TG, and the N^+^-pocket connected with the drain region only exists in NP-EHBTFET and DGNP-EHBTFET. Based on the charge plasma concept [33,34], chromium with a work-function of 4.5 eV [35] is employed as TG to form an electron layer in the left-side channel near TG (i.e., the “N” region in Figure 1), and rhenium formed at a specified pressure and temperature (work-function = 5.5 eV) [36] can be picked as the grounded right-gate (RG) to create a hole layer in the right-side channel near RG (i.e., “P” region in Figure 1), so as to provide conditions for LT parallel to the gate electric field. To enhance electron tunneling and effectively suppress gate leakage and the ambipolar current, a HfO_2_/SiO_2_ heterogate dielectric is adopted in the proposed EHBTFET, which is widely used in TFETs [9,11,14]. Moreover, P-type doping with a concentration of 1 × 10^19^ cm^−3^ is carried out in the source region, while N-type doping with a concentration of 1 × 10^19^ cm^−3^ and 2 × 10^19^ cm^−3^ is performed in the drain region and the N^+^-pocket, respectively. Except for the source, the drain, and the N^+^-pocket regions, the other parts of the channel are intrinsic. In simulations, some key material parameters for In_0.53_Ga_0.47_As—the band gap, heavy-hole effective mass, light-hole effective mass, and electron effective mass—are 0.751 eV, 0.457 *m*_0_, 0.052 *m*_0_, and 0.042 *m*_0_, respectively [30,37].

The Silvaco Atlas 2D numerical simulation platform is used for all device simulations. To more accurately model the tunneling process, the non-local band-to-band (BTBT) tunneling model is adopted, which considers the spatial variation in the energy bands. The Lombardi mobility model with a remote coulomb scattering term is employed to account for the mobility degradation that occurs inside the inversion layers. Considering that the introduction of the HfO_2_ dielectric will generate a large number of traps at the HfO_2_/InGaAs interface, we adopt the trap-assisted tunneling model in simulations, and a single donor trap level is assumed near the valence band with an interface trap density of 1 × 10^13^ cm^−2^ eV^−1^. The limitation of this approach is that it cannot reflect the effect of the midlevel position of traps, which may over- or under-estimate *SS* and *I*_OFF_. In light of the quantum confinement effect in a thin channel layer, we adopt the density gradient model. Additionally, the Shockley–Read–Hall and Auger recombination models, drift-diffusion current transport model, bandgap-narrowing models, Fermi–Dirac carrier statistics, and Schenk oxide-tunneling model are included in the simulations. For these three EHBTFETs, corresponding physical quantities used for revealing some physical mechanisms can be extracted along cutlines A–A’, B–B’, and C–C’ (see dotted lines in Figure 1c), respectively. During data processing, *I*_OFF_ and *I*_ON_ are extracted from the transfer characteristic curves in the OFF-state (*V*_gs_ = 0 V and *V*_ds_ = 0.5 V) and ON-state (*V*_gs_ = 1 V and *V*_ds_ = 0.5 V), respectively.

The proposed DGNP-EHBTFET can be manufactured by state-of-the-art process technology. A brief process flow for obtaining DGNP-EHBTFET is as follows. In stage (1), the In_0.53_Ga_0.47_As epitaxial layer is grown vertically on InP substrate using the molecular-beam epitaxy technique. P^+^-source, N^+^-pocket, and N^+^-drain are formed via ion implantation. Then, the epitaxial layer is etched using reactive ion etching or inductively coupled plasma etching in stage (2). To obtain the required N^+^-pocket and channel width, the key process focuses on the accurate design of the mask pattern. In stage (3), HfO_2_ can be deposited using the atomic layer deposition technique, followed by the etching of dielectrics and metal electrode deposition in stage (4). Finally, SiO_2_ deposition is carried out in stage (5). Moreover, to reduce the interface trap in real operation, good quality of the HfO_2_/InGaAs interface should be possessed, which can be realized through adoption of the Al_2_O_3_/HfO_2_ stack and the assurance of a good manufacturing process [38,39].

## 3. Results and Discussion

### 3.1. Operating Mechanism of Three EHBTFETs

**Figure 2:**
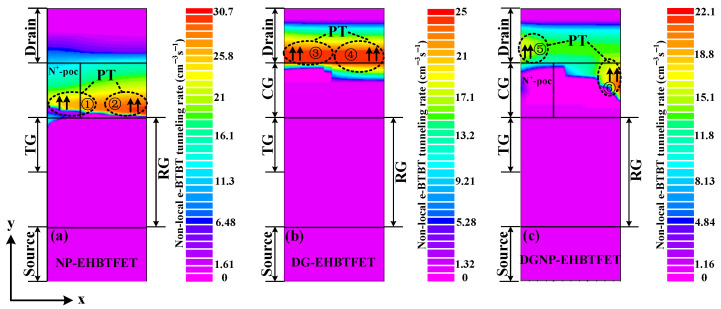
Contour plots of the vertical non-local e-BTBT tunneling rate for (**a**) NP-EHBTFET; (**b**) DG-EHBTFET; and (**c**) DGNP-EHBTFET, in the OFF-state.

**Figure 3:**
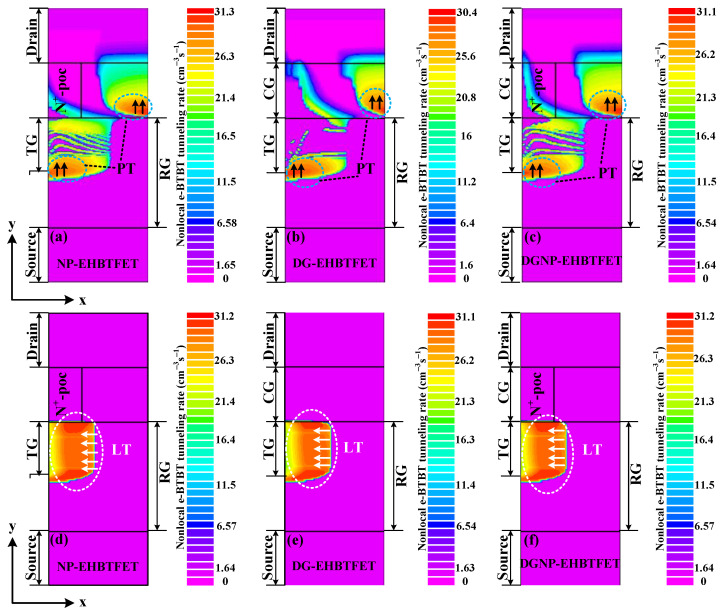
Contour plots of the vertical non-local e-BTBT tunneling rate for (**a**) NP-EHBTFET; (**b**) DG-EHBTFET; and (**c**) DGNP-EHBTFET, in the ON-state. Contour plots of the lateral non-local e-BTBT tunneling rate for (**d**) NP-EHBTFET; (**e**) DG-EHBTFET; and (**f**) DGNP-EHBTFET, in the ON-state.

The operating mechanisms of NP-EHBTFET, DG-EHBTFET, and DGNP-EHBTFET can be interpreted using the non-local e-BTBT tunneling rate. For a clear explanation, we simulate the non-local e-BTBT tunneling rates for these three EHBTFETs from the perspective of vertical tunneling and lateral tunneling, respectively, with the results are shown in Figure 2 and Figure 3. Figure 2 shows the vertical non-local e-BTBT tunneling rate in the OFF-state. It is observed that vertical tunneling (see black arrows) appears above the gate overlap region between TG and RG for NP-EHBTFET (see black dotted circles ➀ and ➁). It is because of the existence of N^+^-pocket that the energy band of this region bends downward, so as to provide conditions for vertical tunneling. As shown in Figure 2b, the CG in DG-EHBTFET can induce holes in its right-side region, which forms a PN junction between this region and the drain region, and eventually results in vertical tunneling in black dotted circles ➂ and ➃. Figure 2c shows that the vertical tunneling of DGNP-EHBTFET mainly occurs in black dotted circles ➄ and ➅, and its non-local e-BTBT tunneling rate is lower than that of NP-EHBTFET and DG-EHBTFET. Thus, it can be seen that the simultaneous introduction of the CG and N^+^-pocket in DGNP-EHBTFET contributes to the suppression of vertical tunneling in the OFF-state. This is mainly due to their regulation of electron and hole concentrations in the gate underlap region near the drain. Since there is no lateral tunneling in these EHBTFETs in the OFF-state, it is concluded that vertical tunneling dominates in this state.

It is observed from Figure 3 that the distribution ranges of vertical tunneling for these three EHBTFETs (see green dotted circles in Figure 3a–c) are basically the same in the ON-state, which is due to the fact that *V*_gs_ applied to TG turns on electron tunneling in the left side of the source and the gate overlap region near RG. As shown in Figure 3d–f, since the TG and RG are the same for these three EHBTFETs, an identical electron–hole bilayer can be induced in the gate overlap region. In the ON-state, electrons tunnel from the valence band of the hole layer into the conduction band of the electron layer under the action of *V*_gs_. Therefore, lateral tunneling (see white arrows in Figure 3d–f) also exists in these three EHBTFETs. Through comparison, we can see that the region available for vertical tunneling is only a few nanometers wide, so can be named PT, while that for lateral tunneling covers the entire gate overlap region (50 nm in length), so can be named LT. Combining the non-local e-BTBT tunneling rate of these two types of tunneling, it follows that LT dominates in the ON-state but PT does in the OFF-state.

### 3.2. Comparison of DC Performance among Three EHBTFETs

**Figure 4:**
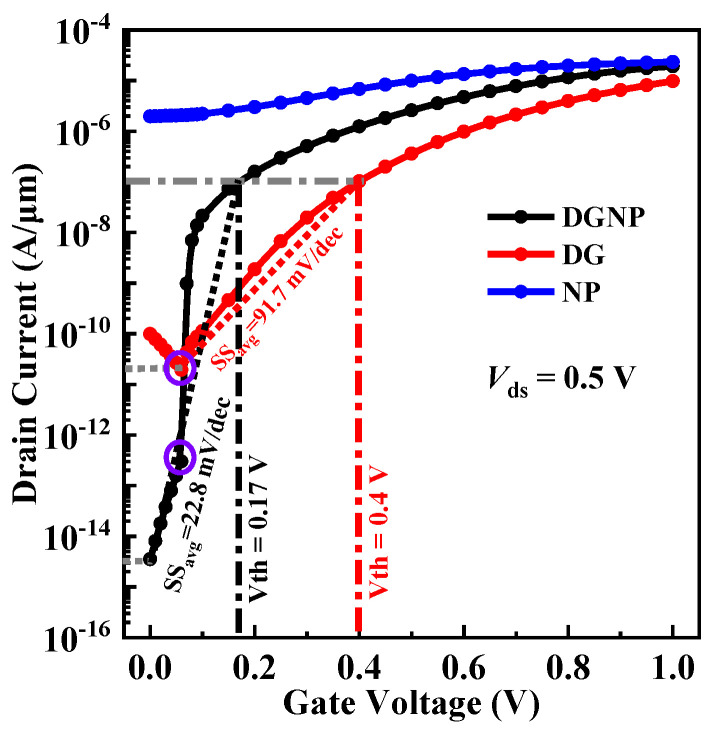
Transfer characteristics of DG-EHBTFET, NP-EHBTFET, and DGNP-EHBTFET.

To compare DC performance, the transfer characteristics of DG-EHBTFET, NP-EHBTFET, and DGNP-EHBTFET are calculated and plotted in Figure 4. It is found from the figure that the *I*_OFF_ of the proposed DGNP-EHBTFET is 3.54 × 10^−15^ A/μm, which is about four and nine orders of magnitude lower than that of DG-EHBTFET and NP-EHBTFET (9.9 × 10^−11^ A/μm and 1.97 × 10^−6^ A/μm), respectively. The *I*_ON_ of DGNP-EHBTFET is close to 1.92 × 10^−5^ A/μm, almost the same magnitude as that of NP-EHBTFET but higher than that of DG-EHBTFET. Therefore, the maximum *I*_ON_/*I*_OFF_ can be obtained by DGNP-EHBTFET, and its value is 5.42 × 10^9^.

The tunneling current depends on the tunneling probability (*P*_tun_), which can be expressed as Equation (1) [14]

(1) $$\begin{array}{ll} P_{\mathrm{tun}}\propto \exp\left(-\frac{4\lambda \sqrt{2m*}E_{g}^{3/2}}{3|e|\hbar (E_{g}+\Delta \varphi )}\right) & =\exp\left(-\frac{4\sqrt{2m*}E_{g}^{3/2}}{3|e|\hbar E}\right)\\ & =\exp\left(-\frac{4\sqrt{2m*}E_{g}^{3/2}}{3|e|\hbar (E_{g}+\Delta \varphi )}\sqrt{\frac{\varepsilon_{\mathrm{sem}}}{\varepsilon_{\mathrm{die}}}t_{\mathrm{sem}}t_{\mathrm{die}}}\right) \end{array}$$

where Δ*φ* is the energy range used for carrier tunneling, *E* is the electric field, m* is the effective carrier mass, *λ* is the tunneling distance, *E*_g_ is the band gap, and *ε*_die_, *ε*_sem_, *t*_die_, and *t*_sem_ are the permittivity and the thickness of the dielectric and the bulk material, respectively. Since *P*_tun_ is closely related to Δ*φ* and *E*, we can interpret the difference between *I*_OFF_ and *I*_ON_ for these three EHBTFETs based on them.

**Figure 5:**
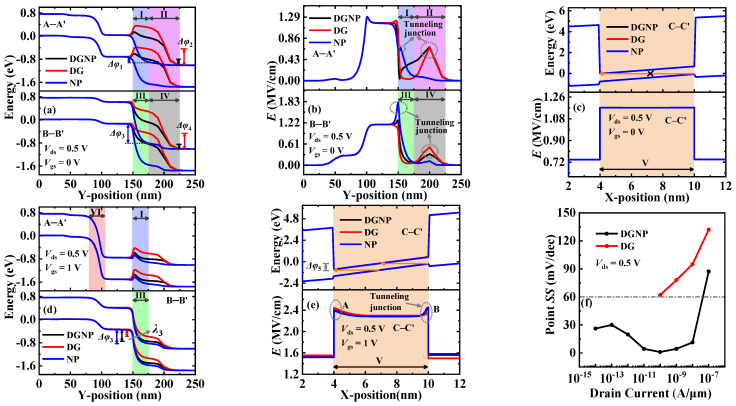
(**a**) Energy band profiles along A–A’ and B–B’ in the OFF-state; (**b**) electric field profiles along A–A’ and B–B’ in the OFF-state; (**c**) energy band and electric field profiles along C–C’ in the OFF-state; (**d**) energy band profiles along A–A’ and B–B’ in the ON-state; (**e**) energy band and electric field profiles along C–C’ in the ON-state; and (**f**) point *SS* versus drain current for EHBTFETs.

Figure 5 shows the energy band and the electric field profiles extracted along cutlines A–A’, B–B’, and C–C, respectively, in the OFF- and ON-states. To explain the physical mechanism more clearly, six regions (i.e., regions I to VI) are defined in the figures of this paper. This is because two kinds of tunneling mechanisms (PT and LT) exist in these three EHBTFETs, which occur in different regions based on different devices and states (see Figure 2 and Figure 3). As shown in Figure 5a, in the OFF-state, PT in NP-EHBTFET occurs in regions I and III, while that in DG- and DGNP-EHBTFETs occurs in regions II and IV. This is because the alignment of the conduction band and the valence band in these regions makes the energy range Δ*φ* (named Δ*φ*_1_ to Δ*φ*_6_ in regions I to VI, respectively) that is available for carrier tunneling exist in regions I and III of NP-EHBTFET and regions II and IV of the other two EHBTFETs, respectively. Actually, the position of Δ*φ* in DG-EHBTFET and DGNP-EHBTFET is different from that in NP-EHBTFET because the introduction of the control-gate CG lifts the energy band near it so as to shift the tunneling junction to the right. Moreover, for DGNP-EHBTFET, the simultaneous introduction of the CG and the N^+^-pocket can optimize its energy band profiles in four regions, which makes it have the narrowest Δ*φ*. For further analysis, the electric field profiles in the OFF-state are calculated and plotted in Figure 5b. Since tunneling cannot occur in the regions without Δ*φ*, we only compare *E* at the tunneling junctions of the four regions (see dotted circles in Figure 5b). It is observed that *E* at the tunneling junction of regions I and II is basically the same, but is very different in regions III and IV, so it can be inferred that the difference in *P*_tun_ mainly depends on *E* in regions III and IV. Obviously, DGNP-EHBTFET possesses the lowest *E* of the tunneling junction. The lower the *E* and the narrower the Δ*φ*, the lower the *P*_tun_ of DGNP-EHBTFET that can be obtained, according to Equation (1). Furthermore, in the OFF-state, the energy band and the electric field profiles based on LT are also calculated, as shown in Figure 5c. It is found that the identical *E*s exist in the region V for these three EHBTFETs, which can be explained by Equation (2), expressed as

(2) $$E=\frac{E_{g}+\Delta \varphi }{\lambda }=\frac{E_{g}+\Delta \varphi }{\sqrt{\frac{\varepsilon_{\mathrm{sem}}}{\varepsilon_{\mathrm{die}}}t_{\mathrm{sem}}t_{\mathrm{die}}}}$$

Since the gate overlap region for these three EHBTFETs has the same dielectric and bulk materials, the same *t*_die_, *t*_sem_, *ε*_die_, *ε*_sem_, and *E*_g_ are possessed in this region. Moreover, Figure 5c shows that there is no Δ*φ* available for LT. According to Equation (2), the same *E* should exist in region V. Although there is *E* in region V, Δ*φ* is absent; thus, no LT appears. It is thus clear that PT is dominant in the OFF-state, eventually resulting in DGNP-EHBTFET with the lowest *I*_OFF_.

To investigate *I*_ON_, the energy band profiles in the ON-state are calculated and shown in Figure 5d. It is seen that PT occurs in regions III and VI simultaneously because the applied *V*_gs_ bends down the energy bands in both regions. Due to the existence of CG, the energy bands of DG-EHBTFET are lifted obviously in regions I and III. The elevated energy band in region I hinders the tunneling electrons drifting to the drain, while that in region III reduces Δ*φ*_3_ and increases *λ*_3_ so as to directly weaken the carrier tunneling, both of which lead to a reduction in the *I*_ON_ of DG-EHBTFET. Since the introduction of the N^+^-pocket can lower the energy bands in regions I and III, a high *I*_ON_ can be maintained in DGNP-EHBTFET like in NP-EHBTFET. In the ON-state, the existence of Δ*φ* (see Δ*φ*_5_ in Figure 5e) enhances the *E* of LT, which is consistent with the result in Equation (2). However, unlike *E* in the OFF-state, *E* in regions A and B (see dotted circles in Figure 5e) in the ON-state further increases. This is because PT also affects the *E* of LT in the gate overlap region. It is observed from Figure 5d that the raised energy band in region I of DG-EHBTFET causes a large number of tunneling electrons from region VI to concentrate in the gate overlap region near TG, eventually resulting in the highest *E* in its region A. However, the existence of the N^+^-pocket bends the energy band downward in region III for NP-EHBTFET and DGNP-EHBTFET, which is more conducive to the accumulation of holes in the gate overlap region near RG, leading to a higher presence of *E* in region B (i.e., tunneling junction) of these two EHBTFETs. The higher the *E*, the higher the *I*_ON_ that can be obtained. Therefore, the *I*_ON_ of three EHBTFETs results in the aforementioned results.

The average subthreshold swing (*SS*_avg_) is obtained using Equation (3) [9,14]

*SS*_avg_ = (*V*_th_ − *V*_OFF_)/(log *I*_Vth_ − log *I*_VOFF_), (3)

where the subthreshold voltage (*V*_th_) represents *V*_gs_ at which the drain current (*I*_ds_) equals 0.1 μA/μm (i.e., *I*_Vth_), *V*_OFF_ is *V*_gs_ at which *I*_ds_ begins to increase with *V*_gs_, and *I*_VOFF_ is the corresponding *I*_ds_ when *V*_gs_ = *V*_OFF_. It is found from Figure 4 that neither the *V*_th_ nor *SS*_avg_ of NP-EHBTFET can be extracted because NP-EHBTFET has been turned on in the OFF-state. Thus, only the values of *SS*_avg_ and *V*_th_ for DG-EHBTFET and DGNP-EHBTFET are compared here. The *V*_th_ and *SS*_avg_ of DGNP-EHBTFET are 0.17 V and 22.8 mV/decade, respectively, which are reduced by 57.5% and 75.1%, respectively, compared with DG-EHBTFET. Moreover, DGNP-EHBTFET exhibits *V*_OFF_ and *I*_VOFF_ values approaching 0 V and 3.54 × 10^−15^ A/μm, respectively, lower than those of DG-EHBTFET. In particular, its *I*_VOFF_ is four orders of magnitude lower than that of DG-EHBTFET, which is because PT, known from the previous analysis, is dominant in the OFF-state. For these two EHBTFETs, LT begins to operate when *V*_gs_ is applied to the left-gate, and its influence on *I*_ds_ starts to exceed PT when *V*_gs_ > 0.06 V (see *I*_ds_ distortion in the purple circles in Figure 4). Since the introduction of the N^+^-pocket can enhance the built-in electric field in the electron–hole bilayer so as to boost the *P*_tun_ of LT, *I*_Vth_ can be achieved under lower *V*_th_ for DGNP-EHBTFET, which is consistent with the results in Figure 4. As a result, a smaller difference between *V*_th_ and *V*_OFF_ and a greater one between *I*_Vth_ and *I*_VOFF_ exists in DGNP-EHBTFET, leading to a lower *SS*_avg_ in it. Point *SS* at each *I*_ds_ is expressed as d*V*_gs_/d(log*I*_ds_). To clearly exhibit the change in *SS*, point *SS* values are extracted from the transfer characteristic curves and plotted in Figure 5f. Note that the point *SS* values of NP-EHBTFET cannot be extracted, and the reason is the same as for *SS*_avg_ extraction. It is observed from the figure that the proposed DGNP-EHBTFET has steeper point *SS* when *V*_gs_ < *V*_th_, and its minimum point *SS* is as low as 1.1 mV/decade. Based on above analyses, DGNP-EHBTFET possesses better DC performance.

### 3.3. Effect of N^+^-Pocket on DGNP-EHBTFET

**Figure 6:**
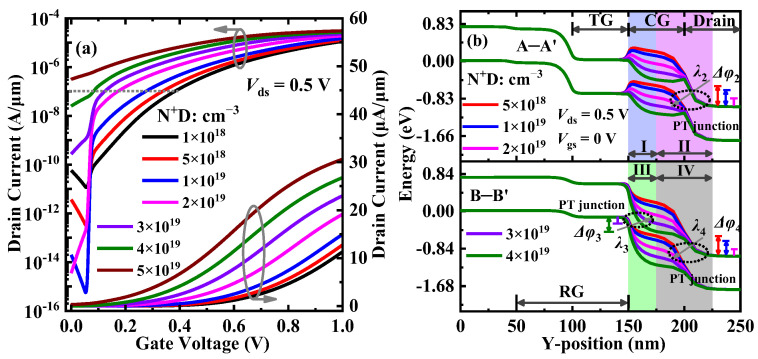
(**a**) Transfer characteristics and (**b**) energy band profiles along A–A’ and B–B’ in the OFF-state, for DGNP-EHBTFET with N^+^D = 5 × 10^18^~4 × 10^19^ cm^−3^.

Figure 6a shows the transfer characteristics of DGNP-EHBTFET with the N^+^-pocket having a different N-type doping concentration (N^+^D). It is found from the figure that *I*_OFF_ decreases first, and then, increases with the increase in N^+^D, and achieves the minimum value at N^+^D = 2 × 10^19^ cm^−3^. To interpret the variation in *I*_OFF_, the energy band profiles in the OFF-state are calculated and shown in Figure 6b. It is observed from Δ*φ* that PT appears in regions II and IV when N^+^D ≤ 2 × 10^19^ cm^−3^ while the same occurs in region III when N^+^D > 2 × 10^19^ cm^−3^. With the increase in N^+^D, both Δ*φ*_2_ and Δ*φ*_4_ decrease but Δ*φ*_3_ increases. Though *P*_tun_ can be reduced by narrowing Δ*φ* to obtain a lower *I*_OFF_, *λ* (named *λ*_1_ to *λ*_6_ in regions I to VI, respectively) should also be taken into account according to Equation (1). Unlike *λ* in region III, those in regions II and IV increase with N^+^D, and their lengths are greater. Hence, one can see that the maximum *λ* and the minimum Δ*φ* simultaneously exist when N^+^D = 2 × 10^19^ cm^−3^, thereby resulting in the minimum *P*_tun_ based on Equation (1), eventually obtaining the minimum *I*_OFF_. In fact, the change in the trend of *I*_OFF_ is dependent on the position of the PT junction (see black dotted circles in Figure 6b). Since CG can induce holes in the top gate underlap region, PN junctions available for tunneling (i.e., the PT junction in regions II and IV) can be created between this region and the drain region. With there is an increase in N^+^D, the increasing electrons in the top gate underlap region bend the energy band downward so as to reduce the aligned energy range for tunneling, eventually degrading *I*_OFF_. When N^+^D > 2 × 10^19^ cm^−3^, the PT junction shifts to region III because a new PN junction is formed by electron accumulation in the top gate underlap region and hole accumulation in the gate overlap region near RG. With the further increase in N^+^D, more electrons gather in the top gate underlap region, which enlarges Δ*φ* so as to promote the electron tunneling. Therefore, *I*_OFF_ exhibits an increasing trend. Moreover, further investigation reveals that there is basically no impact of the variation in N^+^D on LT in the OFF-state. With the increase in N^+^D, *I*_ON_ values exhibit a similar trend of linear increase and are in the same order of magnitude, which is because the electric field at the LT junction is slightly adjusted with the change in N^+^D on the basis of the same electron–hole bilayer. It can also be found from Figure 6a that the lower the *V*_gs_, the greater the influence of N^+^D on *I*_ds_. This is because at low *V*_gs_, LT mainly depends on the built-in electric field in the electron–hole bilayer, which can be enhanced with an increase in N^+^D. A stronger built-in electric field will cause a higher *P*_tun_ of LT so as to turn on DGNP-EHBTFET more easily. Therefore, with the increase in N^+^D, *V*_th_ takes on a linear decreasing trend, except that it cannot be extracted when N^+^D = 5 × 10^19^ cm^−3^. *I*_ON_/*I*_OFF_ exhibits the opposite trend to *I*_OFF_, and approaches the maximum value of 5.42 × 10^9^ at N^+^D = 2 × 10^19^ cm^−3^. Benefiting from the minimum *I*_VOFF_ and the low *V*_th_, the minimum *SS*_avg_ is reached when N^+^D = 2 × 10^19^ cm^−3^. As a result, the optimal device performance can be obtained at N^+^D = 2 × 10^19^ cm^−3^.

**Figure 7:**
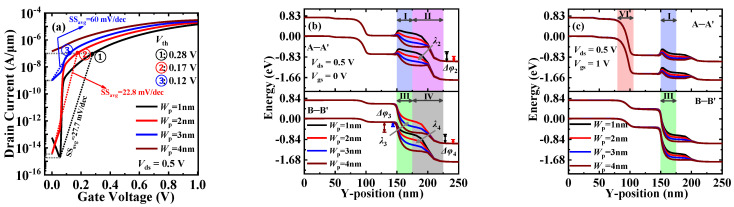
(**a**) Transfer characteristics; (**b**) energy band profiles along A–A’ and B–B’ in the OFF-state; and (**c**) energy band profiles along A–A’ and B–B’ in the ON-state, for DGNP-EHBTFET with *W*_p_ = 1~4 nm.

Next, the influence of the width of the N^+^-pocket (*W*_p_) on the DC performance is studied. Figure 7a shows the transfer characteristic curves of DGNP-EHBTFET at *W*_p_ = 1~4 nm, from which various DC parameters such as *I*_OFF_, *V*_th_, *SS*_avg_, etc., can be extracted. By compromising these extracted parameters, it follows that DGNP-EHBTFET possesses the optimal DC performance at *W*_p_ = 2 nm. Meanwhile, it is found that Figure 7a and Figure 6a have the same change trend in different cases, which is because *W*_p_ and N^+^D have identical mechanisms of influence on DGNP-EHBTFET. For example, it is observed from Figure 7b that PT occurs in regions II and IV when *W*_p_ ≤ 2 nm, while it occurs in region III when *W*_p_ > 2 nm. Similarly, DGNP-EHBTFET is of the maximum *λ* and the minimum Δ*φ* at *W*_p_ = 2 nm, which makes it have the minimum *I*_OFF_ under this condition. As shown in Figure 7c, the energy bands in regions I and III gradually bend downward with the increase in *W*_p_, which promotes the drift of tunneling electrons in region VI and the tunneling of electrons in region III, respectively. However, a slight influence of *W*_p_ on LT is dominant in the ON-state; thus, *I*_ON_ increases with *W*_p_ but has the same order of magnitude.

### 3.4. Effect of Gate Work-Function on DGNP-EHBTFET

**Figure 8:**
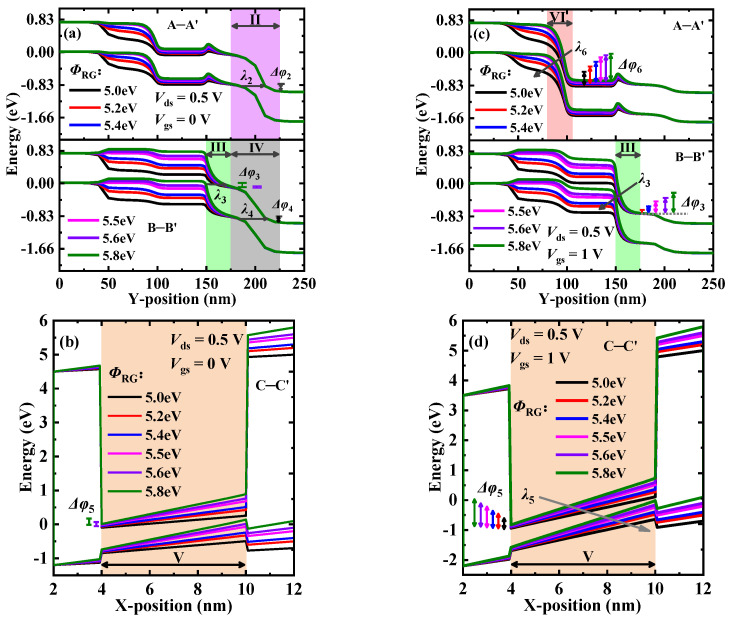
(**a**) Energy band profiles along A–A’ and B–B’ in the OFF-state; (**b**) energy band profiles along C–C’ in the OFF-state; (**c**) energy band profiles along A–A’ and B–B’ in the ON-state; and (**d**) energy band profiles along C–C’ in the ON-state, for DGNP-EHBTFET with *Φ*_RG_ = 5.0~5.8 eV.

Here, we investigate the effect of the work-functions of the right-gate RG, the tunneling-gate TG, and the control-gate CG (i.e., *Φ*_RG_, *Φ*_TG_, and *Φ*_CG_, respectively) on DGNP-EHBTFET, respectively. Table 2 shows the extracted parameters at *Φ*_RG_ = 5.0~5.8 eV. It is found that *I*_OFF_ maintains a relatively low value at *Φ*_RG_ ≤ 5.5 eV, but boosts sharply by six orders of magnitude with the further increase in *Φ*_RG_, which can be seen in the energy band profiles. As shown in Figure 8a, PT occurs in regions II and IV is exactly the same under different *Φ*_RG_ levels. Moreover, because the hole concentration near RG increases with *Φ*_RG_ so as to lift the energy bands in region III, PT can also take place in this region when *Φ*_RG_ > 5.5 eV. Since narrow Δ*φ* and wide *λ* exist in these tunneling regions, the effect of *Φ*_RG_ on PT is insignificant in the OFF-state. Furthermore, it is observed from Figure 8b that LT is opened in region V when *Φ*_RG_ > 5.5 eV. According to the changing trend of *I*_OFF_, it can be concluded that when *Φ*_RG_ > 5.5 eV, the rapid increase in *I*_OFF_ is mainly caused by LT. Figure 8c,d show the energy band profiles in the ON-state regarding PT and LT, respectively. It is seen that with the decrease in *Φ*_RG_, Δ*φ* reduces but *λ* increases in regions III, V, and VI, particularly when *Φ*_RG_ = 5.0 eV, and there is no tunneling in region III. Therefore, *I*_ON_ shows a monotonic decreasing trend with the reduction in *Φ*_RG_ and achieves the minimum value at *Φ*_RG_ = 5.0 eV. Both *SS*_avg_ and *V*_th_ cannot be extracted at *Φ*_RG_ = 5.0 eV as DGNP-EHBTFET is still turned off, even when *V*_gs_ = 1.0 V. Note that parameters that cannot be extracted in this paper are all represented by N/A. Moreover, with the increase in *Φ*_RG_, *I*_ON_/*I*_OFF_ represents a trend of increasing first, and then, decreasing, and *V*_th_ exhibits the opposite trend to *I*_ON_. By comparing these parameters, we assume that DGNP-EHBTFET can maintain better device performance when *Φ*_RG_ = 5.5 eV.

**Figure 9:**
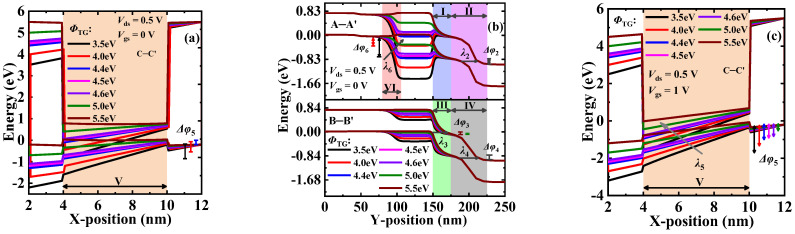
(**a**) Energy band profiles along C–C’ in the OFF-state; (**b**) energy band profiles along A–A’ and B–B’ in the OFF-state; and (**c**) energy band profiles along C–C’ in the ON-state, for DGNP-EHBTFET with *Φ*_TG_ = 3.5~5.5 eV.

Table 3 lists the parameters extracted from the transfer characteristic curves at different *Φ*_TG_ levels. It is found that with the increase in *Φ*_TG_, *I*_OFF_ decreases first, and then, increases, and obtains the minimum value when *Φ*_TG_ = 4.5 eV. To explain in detail, the energy band profiles of LT and PT are calculated. As shown in Figure 9a, when *Φ*_TG_ < 4.5 eV, the accumulation of a large number of electrons near TG opens LT in the OFF-state; thus, the *I*_OFF_ values are much higher than that in other cases. In addition, with the increase in *Φ*_TG_, Δ*φ*_5_ decreases and *λ*_5_ increases, resulting in a gradual reduction in *I*_OFF_. When *Φ*_TG_ ≥ 4.5 eV, *I*_OFF_ is dependent on PT, which makes it have a substantial reduction. Since *Φ*_TG_ just affects the carrier concentration of the gate overlap region (i.e., region V), only the energy bands in this region and the regions connected to it will be adjusted with the change in *Φ*_TG_. It is observed from Figure 9b that the energy bands in region III lift with the increase in *Φ*_TG_, and a new PT appears in this region when *Φ*_TG_ > 4.6 eV; therefore, *I*_OFF_ begins to increase obviously. It is found from Figure 9c that when *Φ*_TG_ < 5.5 eV, LT is dominant in the ON-state, and *I*_ON_ shows a slowly decreasing trend with increasing *Φ*_TG_ based on the variation in Δ*φ*_5_ and *λ*_5_. It is worth noting that only PT exists when *Φ*_TG_ = 5.5 eV, and it appears in regions I and III (not shown). As a result, the minimum *I*_ON_ is obtained at this *Φ*_TG_. Furthermore, the optimal values of *I*_ON_/*I*_OFF_ and *SS*_avg_ can be obtained at *Φ*_TG_ = 4.5 eV. Comprehensive analysis shows that *Φ*_TG_ = 4.5 eV is the best choice for DGNP-EHBTFET.

**Figure 10:**
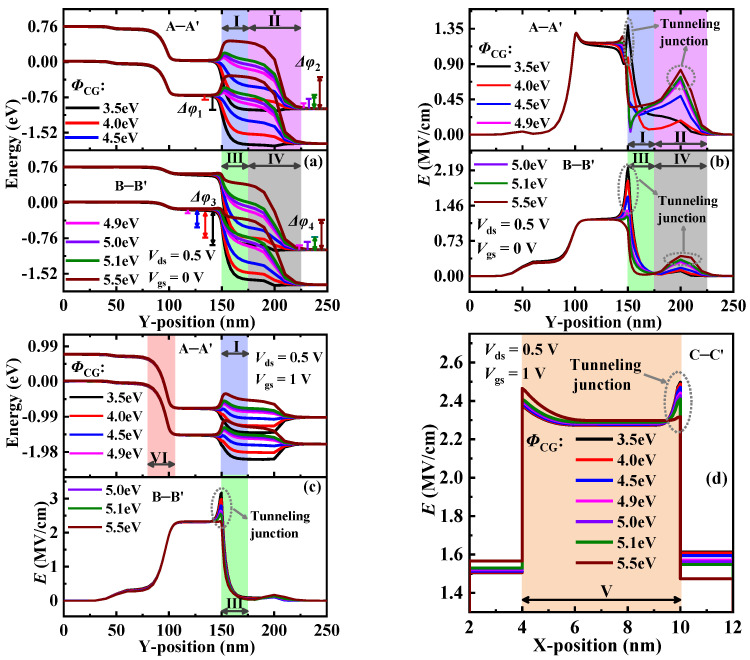
(**a**) Energy band profiles along A–A’ and B–B’ in the OFF-state; (**b**) electric field profiles along A–A’ and B–B’ in the OFF-state; (**c**) energy band profiles along A–A’ and electric field profiles along B–B’, in the ON-state; and (**d**) electric field profiles along C–C’ in the ON-state, for DGNP-EHBTFET with *Φ*_CG_ = 3.5~5.5 eV.

Further, the influence of *Φ*_CG_ on DGNP-EHBTFET is examined, and parameters related to DC performance are extracted and listed in Table 4. As shown in Figure 10a, since the variation in *Φ*_CG_ can adjust the energy band profiles in regions I to IV, the region where PT appears varies with *Φ*_CG_. Because tunneling only exists in the regions with Δ*φ*, we only compare *E* at the tunneling junction to explain the changing trend of *I*_OFF_. As shown in Figure 10b, when *Φ*_CG_ < 4.5 eV, high *E* exists simultaneously at the tunneling junction of regions I and III, which results in a high *I*_OFF_ and turns on DGNP-EHBTFET in the OFF-state. When *Φ*_CG_ > 4.5 eV, *E* at the tunneling junction of regions II and IV increases with *Φ*_CG_, and there is also high *E* at the tunneling junction of region III at *Φ*_CG_ = 4.9 eV. As a result, *I*_OFF_ decreases first, and then, increases, and achieves the minimum *I*_OFF_ at *Φ*_CG_ = 5.0 eV. It is observed from Figure 10c that PT in region VI is insensitive to the change in *Φ*_CG_, but the energy band in region I elevates with the increase in *Φ*_CG_; especially when *Φ*_CG_ > 4.5 eV it begins to hinder the drift of the tunneling electrons, eventually degrading device performance in the ON-state. Moreover, with the increase in *Φ*_CG_, *E* in the tunneling junction of region III gradually weakens, which reduces the *P*_tun_ of PT. As shown in Figure 10d, the varying trend of *E* at the LT junction of region V is the same as that in region III, thereby lowering the *P*_tun_ of LT with increasing *Φ*_CG_. Therefore, *I*_ON_ decreases with the increase in *Φ*_CG_, which is consistent with the results in Table 4. Furthermore, by comparison of the extracted parameters in Table 4, it follows that DGNP-EHBTFET can obtain optimal device performance only when *Φ*_CG_ = 5.0 eV.

## 4. Conclusions

In conclusion, an In_0.53_Ga_0.47_As electron–hole bilayer TFET with a dual-metal left-gate and an N^+^-pocket is proposed and investigated in detail using the Silvaco Atlas 2D numerical simulation platform. The numerical simulations demonstrate that the simultaneous introduction of the control-gate and the N^+^-pocket for the proposed DGNP-EHBTFET can optimize the energy band profiles in the tunneling regions so as to substantially degrade the OFF-state current and maintain good ON-state performance. Further, the impact of the N^+^-pocket on DGNP-EHBTFET is investigated, and the results show that both N^+^D and *W*_p_ focus on the adjustment of the tunneling energy range and the tunneling distance of PT, thereby mainly affecting *I*_OFF_. By compromise of the extracted various parameters, the optimal DC performance can be obtained when N^+^D = 2 × 10^19^ cm^−3^ and *W*_p_ = 2 nm. Considering the change in *Φ*_RG_ and *Φ*_TG_, it is observed that both can control LT and PT through the regulation of the carrier concentration in the overlap region, and comprehensive analysis illustrates that only when *Φ*_RG_ = 5.5 eV and *Φ*_TG_ = 4.5 eV can good device performance be maintained. Furthermore, the investigation shows that the variation in *Φ*_CG_ can not only change the electric field of the tunneling junction, but it can also regulate and control the drift of tunneling electrons. The results show that *Φ*_CG_ = 5.0 eV is the best choice for better DC performance. This paper focuses on revealing the OFF-state leakage suppression mechanism and optimizing the device parameters, without considering the static and dynamic behavior of the proposed DGNP-EHBTFET. In view of their importance, we will put emphasis on this aspect in our next work.

## Figures and Tables

**Table 1:** Device structure parameters used in simulations.

Parameters	Value
Bulk material width (W)	6 nm
N^+^-pocket width (*W*_p_)	2 nm
N^+^-pocket length (*L*_p_)	50 nm
Drain length (*L*_D_)	50 nm
Source length (*L*_S_)	50 nm
Tunneling-gate length (*L*_TG_)	50 nm
Control-gate length (*L*_CG_)	50 nm
Right-gate length (*L*_R__G_)	100 nm
HfO_2_ length (*L*_HfO2_)	150 nm
SiO_2_ length (*L*_SiO2_)	100 nm
Dielectric width near gate (*W*_ox_)	2 nm
Tunneling-gate work-function (*Φ*_TG_)	4.5 eV
Control-gate work-function (*Φ*_CG_)	5.0 eV
Right-gate work-function (*Φ*_RG_)	5.5 eV

**Table 2:** Extracted parameters from transfer characteristic curves at *Φ*_RG_ = 5.0~5.8 eV.

*Φ*_RG_ (eV)	*I*_OFF_ (A/μm)	*I*_ON_ (A/μm)	*I*_ON_/*I*_OFF_	*V*_th_ (V)	*SS*_avg_ (mV/dec)
5.0	1.50 × 10^−19^	1.87 × 10^−8^	1.25 × 10^11^	N/A	N/A
5.2	1.15 × 10^−18^	1.20 × 10^−6^	1.04 × 10^12^	0.52	30.7
5.4	2.59 × 10^−17^	5.79 × 10^−6^	2.24 × 10^11^	0.32	33.4
5.5	3.54 × 10^−15^	1.92 × 10^−5^	5.42 × 10^9^	0.17	22.8
5.6	2.58 × 10^−9^	2.87 × 10^−5^	1.11 × 10^4^	0.14	88.1
5.8	5.16 × 10^−9^	4.30 × 10^−5^	8.33 × 10^3^	0.11	85.4

**Table 3:** Extracted parameters from transfer characteristic curves at *Φ*_TG_ = 3.5~5.5 eV.

*Φ*_TG_ (eV)	*I*_OFF_ (A/μm)	*I*_ON_ (A/μm)	*I*_ON_/*I*_OFF_	*V*_th_ (V)	*SS*_avg_ (mV/dec)
3.5	1.64 × 10^−7^	2.19 × 10^−5^	1.34 × 10^2^	N/A	N/A
4.0	7.87 × 10^−8^	2.11 × 10^−5^	2.68 × 10^2^	0.02	192.3
4.4	7.90 × 10^−9^	1.99 × 10^−5^	2.52 × 10^3^	0.12	108.9
4.5	3.54 × 10^−15^	1.92 × 10^−5^	5.42 × 10^9^	0.17	22.8
4.6	8.51 × 10^−15^	1.81 × 10^−5^	2.13 × 10^9^	0.24	33.9
5.0	1.03 × 10^−13^	9.93 × 10^−6^	9.64 × 10^7^	0.48	80.2
5.5	6.86 × 10^−13^	3.43 × 10^−6^	5.00 × 10^6^	0.51	98.8

**Table 4:** Extracted parameters from transfer characteristic curves at *Φ*_CG_ = 3.5~5.5 eV.

*Φ*_CG_ (eV)	*I*_OFF_ (A/μm)	*I*_ON_ (A/μm)	*I*_ON_/*I*_OFF_	*V*_th_ (V)	*SS*_avg_ (mV/dec)
3.5	1.47 × 10^−5^	4.57 × 10^−5^	3.11 × 10^0^	N/A	N/A
4.0	3.43 × 10^−6^	3.79 × 10^−5^	1.10 × 10^1^	N/A	N/A
4.5	9.13 × 10^−8^	2.90 × 10^−5^	3.18 × 10^2^	0.02	506
4.9	4.39 × 10^−12^	2.13 × 10^−5^	4.85 × 10^6^	0.14	32.1
5.0	3.54 × 10^−15^	1.92 × 10^−5^	5.42 × 10^9^	0.17	22.8
5.1	4.96 × 10^−15^	1.68 × 10^−5^	3.39 × 10^9^	0.23	31.5
5.5	7.04 × 10^−10^	6.37 × 10^−6^	9.05 × 10^3^	0.52	105

## Data Availability

The data presented in this study are available on request from the corresponding author.
